# Peripapillary optical coherence tomography as an alternative to fluorescein angiography for monitoring Behcet’s retinal vasculitis

**DOI:** 10.1038/s41598-021-99485-5

**Published:** 2021-10-08

**Authors:** Mohammad Zarei, Hossein Pesarakli, Mehdi Yaseri, Hamed Etesali, Nazanin Ebrahimiadib

**Affiliations:** 1 Farabi Eye Hospital, Tehran University of Medical Sciences, Tehran, Iran; 2 Department of Epidemiology and Biostatistics, School of Public Health, Tehran University of Medical Sciences, Tehran, Iran; 3 Ocular Immunology and Uveitis Foundation, Waltham, USA

**Keywords:** Retinal diseases, Uveal diseases, Vasculitis syndromes

## Abstract

To study the correlation of OCT parameters including central subfield macular thickness (CSMT), peripapillary retinal thickness (PRT), and peripapillary retinal nerve fiber layer thickness (PNFLT) with fluorescein angiography (FA) in evaluation of inflammatory activity in Behcet’s retinal vasculitis. In this case-series, concurrent FA and OCT were performed. A scoring system was devised for FA. PNFLT in 3.4-mm-diameter circle as well as PRT in doughnut shaped regions between the 1-mm- and 2.2-mm-diameter and between the 2.2-mm- and 3.45-mm-diameter circles was measured. The correlation of FA and OCT parameters was analyzed. A total of 105 sets of FA from 28 eyes (15 patients) were reviewed. Four (26.6%) were female and mean age was 31.6 ± 8.49 years. Each micron increase in CSMT, PRT2.2, PRT3.45, and PNFLT, caused a rise of 0.018 (95% CI 0.008–0.027, P < 0.001, r = 0.413), 0.053 (95% CI 0.035–0.070, P < 0.001, r = 0.443), 0.086 (95% CI 0.065–0.108, P < 0.001, r = 0.707), and 0.185 (95% CI 0.152–to 0.218, P < 0.001, r = 0.850) unit in FA score, respectively. Parameters having significant correlation with angiographic inflammatory activity, were CSMT, PRT2.2, PRT3.45 and RNFLT. Those with the strongest correlation, PRT3.45 and PNFLT, may be considered as quantitative non-invasive alternatives to FA for monitoring Behcet’s retinal vasculitis.

## Introduction

Behcet’s disease is one of the most commonly reported noninfectious uveitis entities in Asia, Middle East and Mediterranean region. Retinal vasculitis, the most devastating manifestation of Behcet’s uveitis, is the leading cause of ocular morbidity and blindness^1^. Considering the poor prognosis of inadequately treated Behcet’s retinal vasculitis, using a sensitive method to monitor the severity of ocular inflammation is necessary for optimal management. Fluorescein angiography (FA) is usually considered the gold standard modality for this purpose. However, the invasive nature of FA and the need for frequent monitoring of inflammation for optimal management, make the finding of a non-invasive alternative modality desirable. In this regard, attempts have been made to investigate the use of laser flare photometer^2^. However, this technique needs specialized machinery that may restrict its widespread use. In recent years, optical coherence tomography has increasingly become a constant feature in routine ophthalmic care. Considering that optic disc hyperfluorescence or leakage in FA is a common sign for intraocular inflammation, evaluation of peripapillary retinal thickness with OCT may have the potential to be used as an index for inflammatory activity. We have already shown that despite normal clinical and even angiographic appearance of the optic disc, peripapillary retinal thickness is increased in Fuchs uveitis^3^. Based on this background, we decided to evaluate the potential of measurement of peripapillary retinal thickness as a non-invasive alternative of FA for monitoring the inflammatory activity in Behcet’s retinal vasculitis.

## Results

Twenty-eight eyes from 15 patients with Behcet’s disease and retinal vasculitis were included in the study. Mean age was 31.6 ± 8.49 years (median = 34 years, range 18–48 years). There were four (26.6%) females and the number of the right eyes was 14 (50%). Mean baseline VA was 0.44 ± 0.57 logMAR (median = 0.18, range 0.00–2.00).

Mean follow up duration was 4.76 ± 3.24 months (median = 5.25 months, range 0–10.5 months) and mean number of imaging sessions was 3.75 ± 2.01 (median = 3.50, range 1–7) (Table 1).

**Table 1:** Number of available image-sets for each imaging modality.

Type of imaging	Number of included image sets
FA	105
Macular OCT	92
Peripapillary retinal OCT (Optic nerve head OCT)	95
Peripapillary retinal nerve fiber layer OCT	97

**Figure 1:**
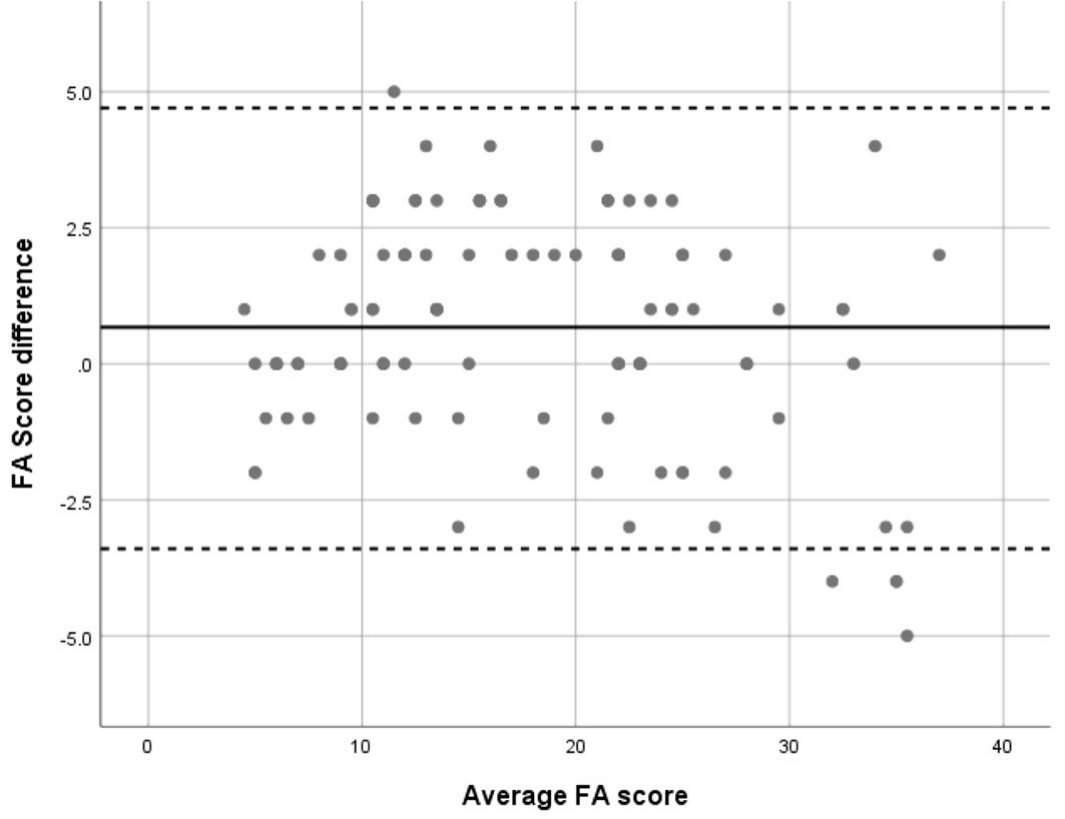
Bland–Altman plot: Bland–Altman plot shows the agreement of inflammatory scores given by two raters.

Mean difference between FA inflammatory scores given by two raters was 0.67 ± 2.07 (median = 1, range − 5 to + 5). Interclass correlation coefficient between FA inflammatory scores of two raters was 0.986 (95% Confidence Interval 0.979–0.990). To demonstrate the agreement between two raters, Bland–Altman plot was used (Fig. 1).

The mean FA inflammatory score was found to be 17.9 ± 8.6 (median = 16, range 4.5–37).

Mixed model analysis and calculation of Spearman’s rho coefficient were used to study correlation between OCT parameters and FA inflammatory scores. It was revealed that every one-micron-rise in CSMT, correlates with a 0.018 (95% CI 0.008–0.027, P < 0.001, r = 0.413) unit increase in FA inflammatory score.

Every one micron-rise in PRT 2.2 and PRT 3.45, was correlated with a 0.053 (95% CI 0.035–0.070, P < 0.001, r = 0.443) and 0.086 (95% CI 0.065–0.108, P < 0.001, r = 0.707) unit increase in FA inflammatory score, respectively.

**Figure 2:**
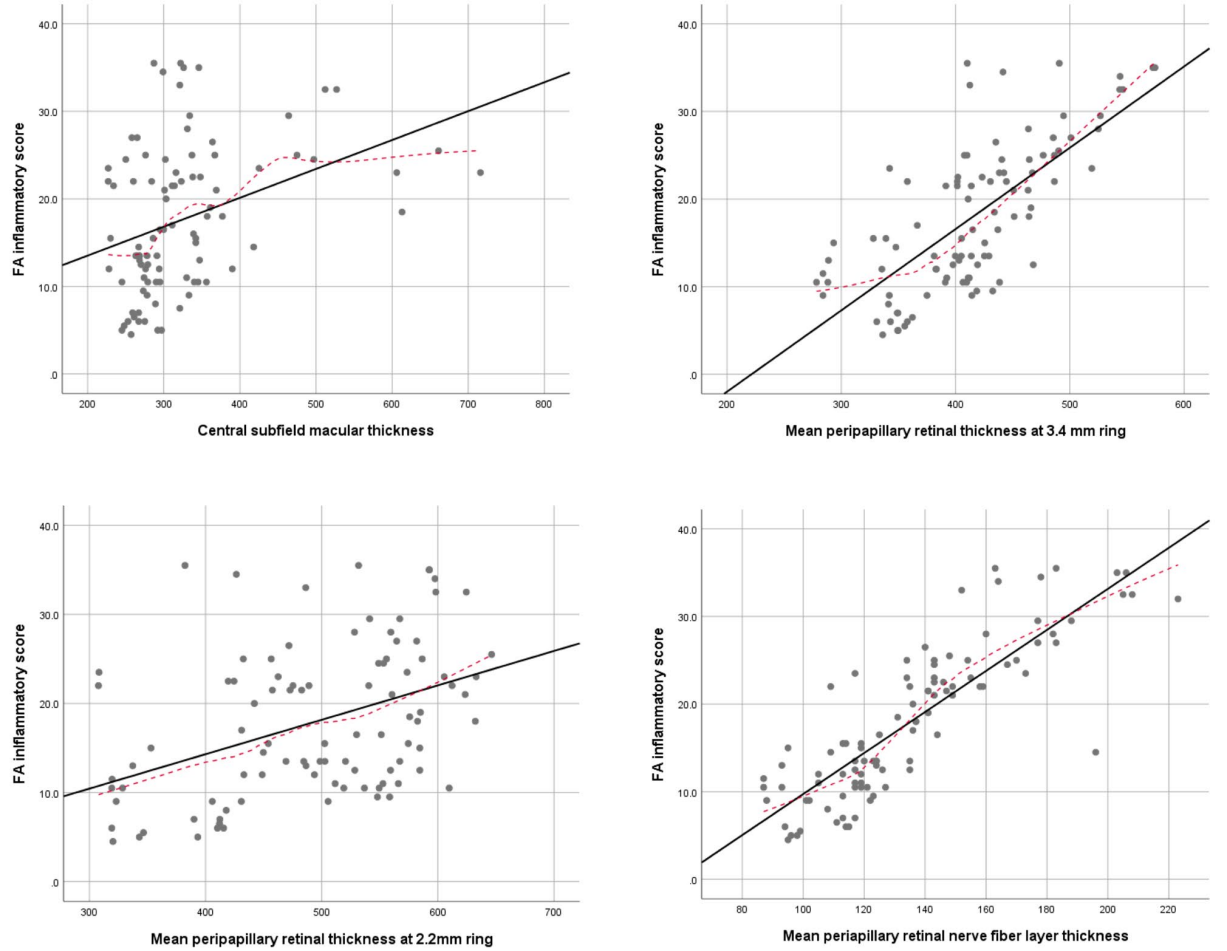
Scatter plot of OCT parameters and FA score: correlation of four OCT parameters with FA inflammatory score in Behcet’s retinal vasculitis.

Every one micron-rise in PNFLT, was correlated with a 0.185 (95% CI 0.152–0.218, P < 0.001, r = 0.850) unit increase in FA inflammatory score (Fig. 2).

To compare these correlation coefficients with each other, the method presented by Hittner et al.^4^ was employed; there was a statistically significant difference between each pair of the Spearman’s rho coefficients (all P-values < 0.001).

A statistically significant correlation was observed between VA and FA inflammatory score, such that a 0.02 logMAR rise was present for each unit increase in FA inflammatory score (95% CI [0.015, 0.030], P < 0.001). A statistically significant correlation was also seen between VA and mean PNFLT; for each micron increase in PNFLT, there was an 0.008 increase in logMAR (95% CI [0.006, 0.011], P < 0.001).

There was no statistically significant correlation between VA and mean PRT 2.2 thickness (P = 0.515), mean PRT 3.45 thickness (P = 0.201) and CSMT (P = 0.062).

## Discussion

Although clinical exam is commonly used for monitoring the severity of inflammation, this approach has several inherent limitations. Despite attempts made to standardize the reporting of inflammatory clinical findings^5^, this method is heavily examiner-dependent and subjective in nature which may lead to inter- and intra-observer variations, especially in case of suboptimal examination skills and expertise. On the other hand, existence, degree and extent of retinal vasculitis, as the most significant vision threatening finding in Behcet’s uveitis, can be readily underestimated in clinical examination. Although FA is the modality of choice to determine the presence, severity, and extent of retinal vasculitis, the qualitative nature of information provided by this modality may interfere with a standardized interpretation and comparison. In this context, obtaining quantitative data reflecting the degree of inflammation is highly desirable. Another drawback of FA is its invasive nature.

Compared to FA, laser flare photometer has the clear advantage of objectivity through providing quantitative data, as well as being a non-invasive method. It has been shown that laser flare photometry is a valid predictor for the grade of inflammation in the posterior segment of the eye which reduces the need for FA^2^. However, this method needs specialized machinery that may have a role in limiting its widespread application in eye clinics. On the other hand, in recent years, OCT has been steadily gaining popularity in routine ophthalmology practice as a non-invasive reliable technology for evaluation the eye, particularly in the evaluation of posterior segment and optic nerve. Nowadays, OCT machines are considered routine equipment of average ophthalmic clinics in many parts of the world. Therefore, it seems highly desirable to find ways to use the already available technology of OCT for monitoring inflammatory activity in Behcet’s retinal vasculitis. It has been already shown that peripapillary retinal thickness increases in uveitis entities even in the absence of clinical or angiographic involvement of optic nerve head^3^. Based on this background, we decided to investigate the correlation between FA-derived inflammatory activity (as the present gold standard method) and OCT retinal parameters to evaluate the potential application of OCT as a surrogate of FA in monitoring inflammation in Behcet retinal vasculitis.

To develop a specialized FA scoring system, we started with previously proposed systems and modified them based on our clinical experience with patients with Behcet’s disease^6–9^. Considering the heterogeneity of angiographic signs in various ocular inflammatory diseases, we think that instead of a “one size fits all” approach, devising a scoring systems tailored for each category of uveitis entities (e.g., retinal vasculitis), or even for specific uveitis diseases (e.g., Behcet’s retinal vasculitis, Harada or sympathetic ophthalmia) may be a better approach.

We tried to devise an FA inflammatory scoring system to reflect the routine application of FA in clinical practice with these patients while maximizing the objectivity. The hallmark of Behcet’s retinal vasculitis is the leakage from retinal capillaries and severity of fluorescein leakage from capillaries is one of the most useful inflammatory indices to monitor inflammation in Behcet’s uveitis. In clinical practice with Behcet patients, posterior angiographic capillary leakage is considered more alarming than peripheral capillary leakage. Compared to peripheral capillary leakage, posterior capillary leakage occurs in more severe cases. Therefore, our proposed scoring system gives more weights to capillary vascular leakage vs leakage from large retinal vessels and to posterior capillary involvement vs peripheral capillary involvement.

Although significant, the FA inflammatory score was found to have the weakest correlation with CSMT (r = 0.413). This may reflect that compared to edema of other regions of the retina (e.g., perifoveal or peripapillary regions), central macula thickening is a later consequence in Behcet retinal vasculitis and occurs in the more severe cases. Therefore, as an inflammatory index, central macular thickness is less sensitive than peripapillary retinal thickness.

Studies from Korea, Japan, Tunisia, Spain and China reviewing FA of patients with Behcet’s disease, report a frequency of about 52–82% for concurrent involvement of optic nerve head with retinal vasculitis^1^. Regarding the strong correlation we found between peripapillary retinal thickness and FA inflammatory scoring, it is interesting that the correlation between FA inflammatory score and peripapillary retinal thickness was stronger for the 3.45-mm ring (r = 0.707) compared to 2.2-mm ring (r = 0.443). It can be speculated that inflammatory edema may affect the peripheral peripapillary retina more readily than its posterior neighborhood. Among OCT parameters, the strongest correlation with FA inflammatory score belonged to 3.4-mm circle PNFL thickness (r = 0.850), which was even stronger than the correlation with 3.45-mm ring peripapillary total retinal thickness (r = 0.707). This may hint that the primary focus of inflammation in Behcet’s retinal vasculitis is inner retina rather than outer retina or choroid, because the inflamed leaky vessels are mostly located in the inner two-thirds of the retina.

There are several limitations for this study. First, subjective and qualitative nature of FA-derived information regarding inflammation, imposes an inherent limitation to attempts in devising any FA scoring system. We tried to mitigate this problem by increasing the number of possible grades which can be attributed to the FA inflammatory findings, especially to findings which are considered the most important in clinical practice (i.e., capillary leakage and macular leakage). We also tried our best to objectify the FA-derived subjective findings. However, due to aforementioned inherent limitations of FA, we expect that not all the FAs with the same inflammatory score, have exactly the same severity of inflammation. In fact, these limitations were among the primary motives for us to seek for a more objective and quantitative method to monitor inflammation. Second, although increased vascular permeability and subsequent edema is a cardinal manifestation of inflammation, vascular occlusions and subsequent retinal nonperfusion are among characteristic features of Behcet’s disease. In such cases, the combination of ischemia and edema complicates the interpretation of retinal thickness measurement as an index of inflammatory activity. It should be noted that in some patients with occlusive retinal vasculitis, concurrent presence of partial ischemia and leakage/edema in the same retinal area, not only interfere with the OCT interpretation, but also makes the interpretation of FA challenging. On the other hand, although “new” areas of capillary non-perfusion may be considered as a marker of active inflammation^8^, there is not always a certain way to differentiate an old area of capillary non-perfusion from a new one in the first imaging session. Therefore, we decided to exclude eyes with significant areas of capillary non-perfusion from this study. This means that our proposed approach to measure the peripapillary retinal thickness as an index of inflammatory activity, may not be applicable to patients with extensive areas of retinal ischemia. This also means that this method might be even more useful in other types of retinal vasculitis which are less occlusive in nature, a hypothesis that is to be addressed in future studies.

It is noteworthy that conventional format for the OCT data output and printouts of peripapillary RNFL thickness measurements are set to highlight the “thinning” not the “thickening”, as their primary purpose is to bring the atrophy (e.g., glaucoma), not edema, to attention. This means that in a regular printout of these OCT machines, there is no color-coding to differentiate a thickened PNFL from a normal-thickness PNFL.

More longitudinal studies are needed to validate the usefulness of our method in monitoring inflammatory activity and guiding treatment, not only in Behcet’s retinal vasculitis but in retinal vasculitides of other etiologies and even in those uveitis entities that are not strongly associated with retinal vasculitis such as JIA.

In conclusion, this study proposes the measurement of peripapillary retinal thickness indices to monitor inflammatory activity of Behcet’s retinal vasculitis. In this method, less invasive and more available OCT imaging systems is employed instead of standard FA. Although for establishing the diagnosis of retinal vasculitis and evaluation of its extent, baseline FA may be necessary, the suggested OCT inflammatory indices can be helpful in reducing the sessions of FA acquisition.

## Method

The study was approved by the Ethics Committee of Tehran University of Medical Science and all investigations adhered to the tenets of the Declaration of Helsinki. Informed consent was obtained from each participant. Patients with Behcet’s disease and angiographically proven retinal vasculitis were included in the study. Diagnosis of Behcet’s disease was established according to International Criteria for Behcet’s Disease^10^. All subjects underwent a complete ophthalmic evaluation, including best corrected visual acuity (BCVA) testing, slit-lamp exam, intraocular pressure (IOP) measurement, and fully dilated fundus examination. Treatment plan of patients was not changed for the study. Patients were excluded if they were pregnant or had diabetes, history of neurologic disorders, history of any intraocular intervention or ocular trauma within three months prior to imaging, capillary non-perfusion with cumulative area greater than 10-disc area on FA, clinical or paraclinical optic atrophy, glaucoma or IOP ≥ 22 mmHg, axial length > 25 mm and < 22 mm, or (if phakic) refractive errors ≥  + 3.00 or ≤  − 3.00 D.

**Figure 3:**
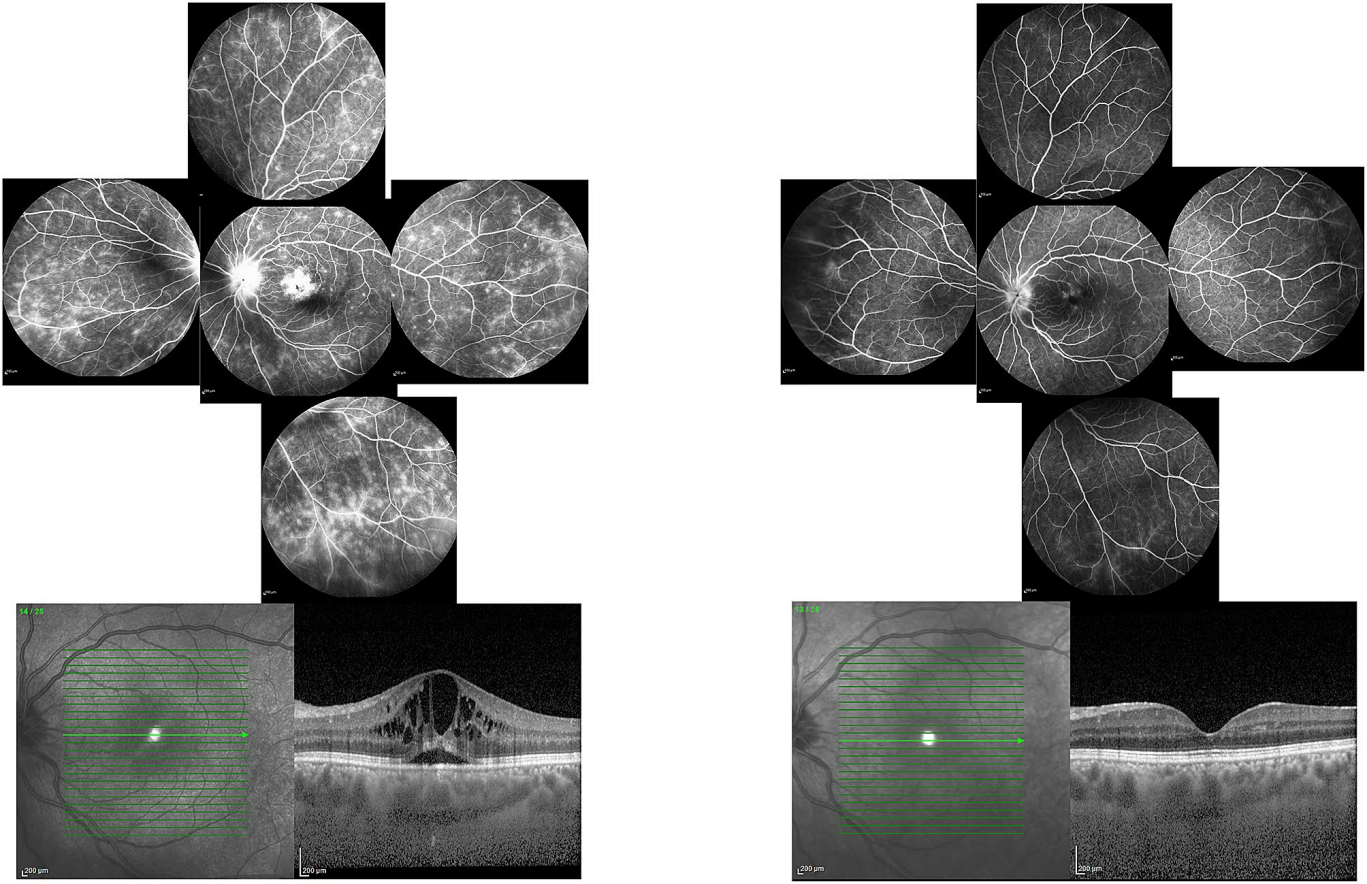
Representative baseline and follow up FA and OCT imaging: concurrent fluorescein angiography (FA) and OCT at baseline (left side) and 10.5 months later (right side) of the left eye of one of our patients with Behcet’s retinal vasculitis. FA is scored 25.5 at baseline and 12.5 at 10.5 months follow up. Both angiographic inflammatory activity in FA (top) and retinal thickness at the foveal area in OCT (bottom) have decreased substantially at 10.5 months follow up compared to baseline.

All patients underwent concurrent OCT imaging (Specteralis, HEYEX software 6.0 Heidelberg Engineering, Heidelberg, Jena, Germany) and FA (Heidelberg Engineering, Heidelberg, Jena, Germany). All imaging sessions with FA and at least one of three types of OCT imaging (macular OCT, optic nerve head OCT (to measure peripapillary retinal thickness), and peripapillary nerve fiber layer OCT) were included. Consecutive imaging sessions from patients with multiple imaging sessions, were included in the study as separate image sets if they were at least 1.5 month apart (Fig. 3).

All images were obtained after pharmacologic mydriasis.

The following guideline was observed for performing FA: after injection of 3–4 cc of 10% sodium fluorescein intravenously, early images of posterior pole of both eyes were captured during the first minute. Next, peripheral sweeps were obtained and then, after about at least 4–5 post-injection minutes, late images of posterior pole were captured. A specialized FA scoring system was developed for this study with minimum and maximum possible inflammatory scores of 0 and 43, respectively (Table 2 and see Supplemental Digital Content 1, which demonstrates examples of FA for various grades in different categories of FA scoring).

**Table 2:** Fluorescein angiography scoring system for grading of inflammatory activity in Behcet’s retinal vasculitis.

**Optic disc HF (maximal score: 3)**
0: normal fluorescence and normal staining of the scleral rim
1: partial staining of the disc
2: diffuse leakage without blurring of the disc margin
3: diffuse leakage and blurring of the disc margin
**Macular HF (maximal score: 4)**
0: no perifoveal hyperfluorescence
1: incomplete ring of leakage
2: complete (360°) leakage but less than 1 DD wide
3: complete (360°) leakage of 1 to 1.5 DD wide
4: complete (360°) leakage of more than 1.5 DD wide
**Large retinal vessel wall HF (proximal to the third bifurcation in the posterior view) (maximal score: 3)**
0: none
1: focal
2: more extended or multifocal HF
3: diffuse HF
**Posterior capillary fluorescein leakage; macular HF should *not* be included (maximal score:10)**
0: none
2: increased visibility of the smallest capillaries or scattered faint capillary leakage
4: diffuse mild capillary leakage
6: more intense diffuse leakage with clear distinction between adjacent vascular domains
8: greater leakage with blending of adjacent leaking domains into each other in less than half of the area of the posterior view (excluding macular HF)
10: greater leakage with blending of adjacent leaking domains into each other in more than half of the area of the posterior view (excluding macular HF)
**Peripheral capillary fluorescein leakage; scoring should be separately done for each quadrant (maximal score for each quadrant: 5, maximal score for four quadrants: 20)**
0: none
1: increased visibility of the smallest capillaries or scattered faint capillary leakage
2: diffuse mild capillary leakage
3: more intense diffuse leakage with clear distinction between adjacent vascular domains
4: greater leakage with blending of adjacent leaking domains into each other in less than half of the area of the peripheral quadrant
5: greater leakage with blending of adjacent leaking domains into each other in more than half of the area of the quadrant
**Hazy media based on posterior view (only if attributable to aqueous or vitreous cells and flare) (maximal score: 3)**
0: indicates clear view of retinal capillaries
1: a dull view of capillaries is appreciated
2: unable to see retinal capillaries (partially or totally) but all larger vessels are visible
3: unable to see larger retinal vessels (partially or totally)
Sum (maximal score: 43)

*HF* hyperfluorescence, *DD* disc diameter.

For the purpose of scoring, the latest available frame of the angiogram in any given region of the retina was selected. Two raters (M.Z. and N.E.) scored the FAs, independently. Then FA sets with maximal inter-rater differences were determined and reviewed by both raters. There were five sets with 6-score difference and one set with 7-score difference. Four weeks after this joint sitting, the raters repeated and finalized their scoring for all FA image sets separately. The mean FA scoring of the two raters was used for analysis.

Segmentation of OCT images were checked and corrected manually if necessary, by M.Z.

Central subfield macular thicknesses (CSMT) were extracted from macular OCTs.

**Figure 4:**
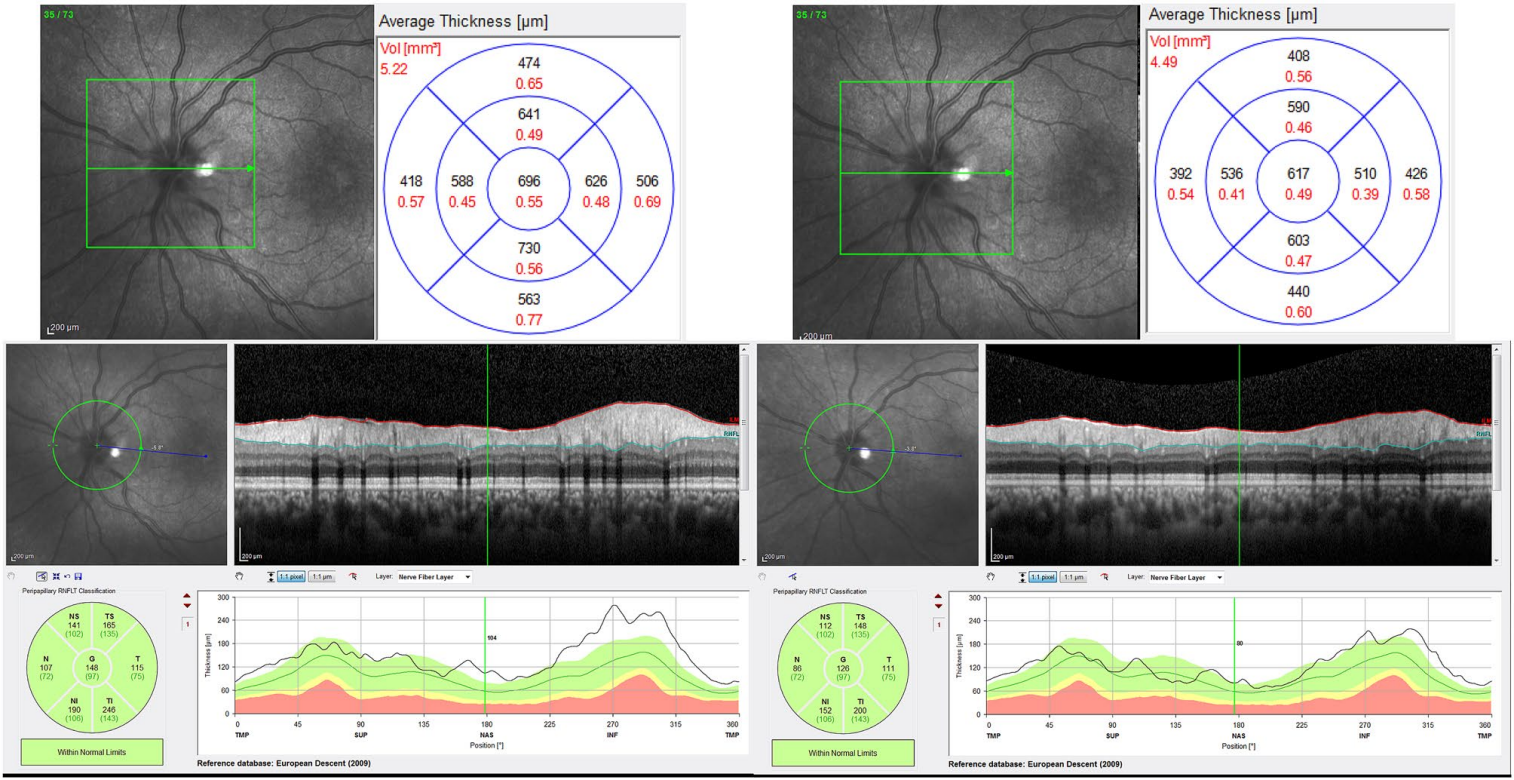
Representative baseline and follow up peripapillary imaging: peripapillary retinal thickness (top) and peripapillary retinal nerve fiber layer thickness (bottom) measurements of the left eye of one of our patients with Behcet’s retinal vasculitis at baseline (left side), and 10.5 months later (right side) show that thickness values approach normal with treatment at 10.5 months follow up time.

Peripapillary retinal thickness (PRT) were measured by ONH raster scan protocol as follows: the ONH raster generated 73 slices (B-scans) over a 4 × 4 × 2 mm^3^ volume centered on the optic nerve head. Circular lines on the cube represent 1-, 2.2-, and 3.45-mm diameter (Fig. 4, top).

To evaluate the inner PRT, the retinal thickness of the doughnut-shaped region between the 1-mm- and 2.2-mm-diameter circles was measured in four quadrants (superior, inferior, temporal, and nasal). To evaluate the outer PRT, the retinal thickness of the doughnut-shaped region between the 2.2-mm- and 3.45-mm-diameter circles was measured in four quadrants (superior, inferior, temporal, and nasal). At the end, average PRT in inner and outer rings was used as the global indices of inner and outer peripapillary retinal thicknesses (PRT 2.2 and PRT 3.45, respectively).

The measurement of the peripapillary retinal nerve fiber layer thickness (PNFLT) was performed using the standard 360°, 3.4 mm diameter circular scan and the PNFL thickness in six sectors as well as mean PNFLT were recorded (Fig. 4, bottom). Mean PNFLT was used for analysis.

### Statistical analysis

Bland–Altman plot and interclass correlation coefficient were used to study the inter-grader agreement between two FA graders.

Mixed model analysis was used to test the correlation between FA inflammatory score and OCT parameters, between FA inflammatory score and VA, and between VA and OCT parameters.

To estimate the correlation between FA inflammatory score and OCT parameters, Spearman’s rho coefficient was calculated.

To compare the calculated correlations (Spearman’s rho coefficients) with each other, the method presented by Hittner et al. was employed^4^.

*P* values less than 0.05 were considered significant.

## Supplementary Information

Supplementary Figure 1.
