# Supplementary material for: Peripapillary optical coherence tomography as an alternative to fluorescein angiography for monitoring Behcet’s retinal vasculitis
Source: Sci Rep. 2021 Oct 8;11:20037. doi: 10.1038/s41598-021-99485-5 (PMC8501075; doi:10.1038/s41598-021-99485-5)
Supplement: Supplementary file 1 — Supplementary Figure 1. [file 41598_2021_99485_MOESM1_ESM.pdf]

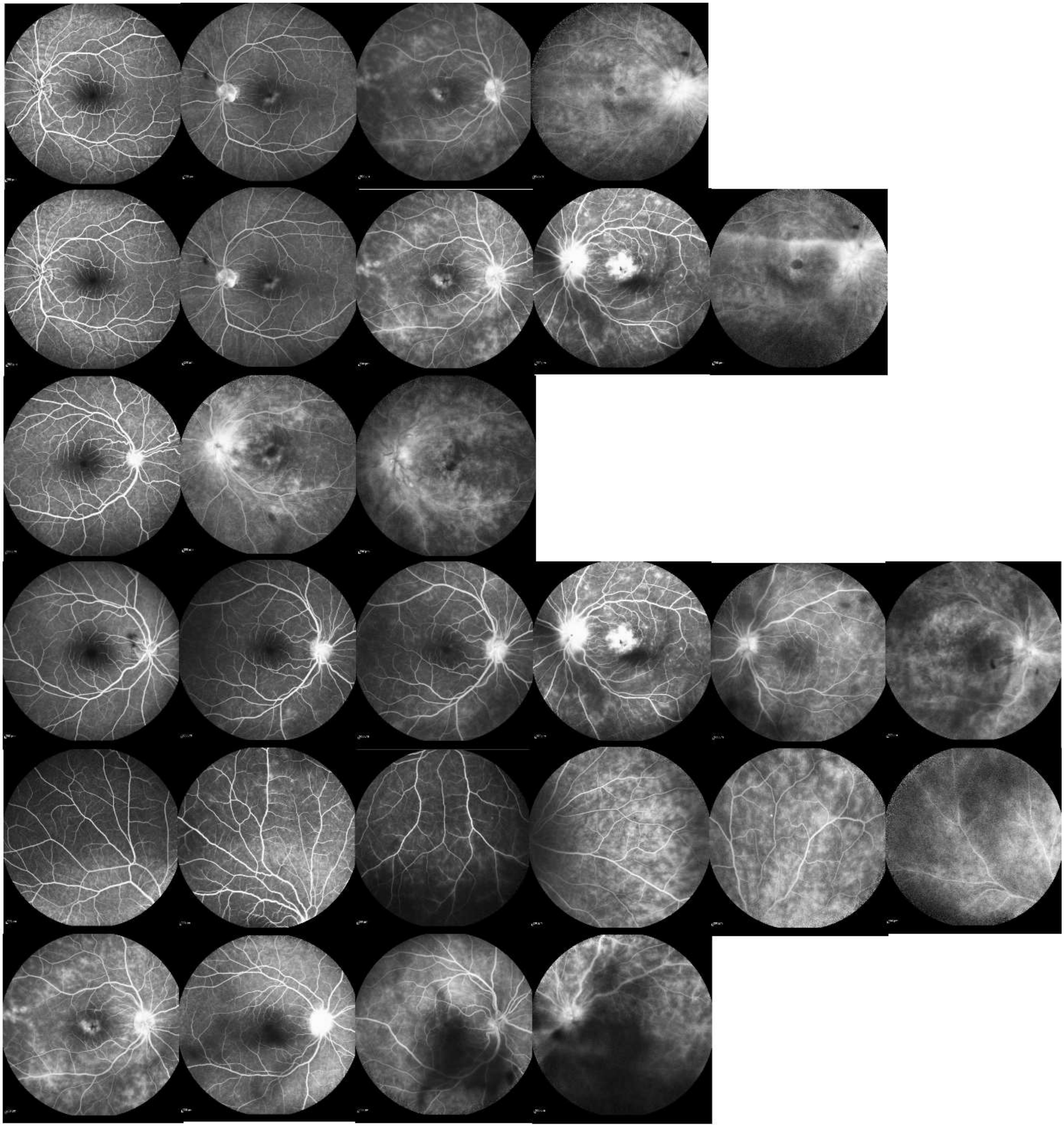

Supplemental Digital Content 1: Fluorescein angiography scoring system for grading of inflammatory activity in Behcet's retinal vasculitis; The prototype of FA for each grade in various categories; First row: **optic disc hyperfluorescence**, scores from left to right (0: normal fluorescence and normal staining of the scleral rim, 1: partial staining of the disc, 2: diffuse leakage without blurring of the disc margin, 3: diffuse leakage and blurring of the disc margin. Secnod row: **Macular hyperfluorescence**, scores from left to right (0: no perifoveal hyperfluorescence, 1: incomplete ring of leakage, 2: complete (360°) leakage but less than 1 disc diameter (DD) wide, 3: complete (360°) leakage of 1 to 1.5 DD wide, 4: complete (360°) leakage of more than 1.5 DD wide). Third row: **Large retinal vessel wall hyperfluorescence** (proximal to the third bifurcation in the posterior view), scores from left to right: (0: none, 1: focal, 2: more extended or multifocal hyperfluorescence). Fourth row: **Posterior capillary fluorescein leakage**; macular hyperfluorescence was *not* included, scores from left to right: (0: none, 2: increased visibility of the smallest capillaries or scattered faint capillary leakage, 4: diffuse mild capillary leakage, 6: more intense diffuse leakage with clear distinction between adjacent vascular domains, 8: greater leakage with blending of adjacent leaking domains into each other in less than half of the area of the posterior view (excluding macular hyperfluorescence), 10: greater leakage with blending of adjacent leaking domains into each other in more than half of the area of the posterior view (excluding macular hyperfluorescence)). Fifth row: **Peripheral capillary fluorescein leakage**; scoring should be separately done for each quadrant, scores from left to right: (0: none, 1: increased visibility of the smallest capillaries or scattered faint capillary leakage, 2: diffuse mild capillary leakage, 3: more intense diffuse leakage with clear distinction

between adjacent vascular domains, 4: greater leakage with blending of adjacent leaking domains into each other in less than half of the area of the peripheral quadrant, 5: greater leakage with blending of adjacent leaking domains into each other in more than half of the area of the quadrant). Sixth row: **Hazy media based on posterior view** (only if attributable to aqueous or vitreous cells and flare), scores from left to right: (0: indicates clear view of retinal capillaries, 1: a dull view of capillaries is appreciated, 2: unable to see retinal capillaries (partially or totally) but all larger vessels are visible, 3: unable to see larger retinal vessels (partially or totally))
